# Factors in the psychosocial work environment of staff are associated with satisfaction with care among older persons receiving home care services

**DOI:** 10.1111/hsc.14045

**Published:** 2022-09-26

**Authors:** Anne‐Marie Boström, Dan Lundgren, Zarina Nahar Kabir, Ingemar Kåreholt

**Affiliations:** ^1^ Division of Nursing, Department of Neurobiology, Care science and Society Karolinska Institutet Stockholm Sweden; ^2^ Theme Inflammation and Aging, Karolinska University Hospital Huddinge Sweden; ^3^ R&D unit, Stockholms Sjukhem Stockholm Sweden; ^4^ Department of Quality and Development, Division of Social Services Värnamo Municipality Värnamo Sweden; ^5^ Institute of Gerontology, Aging Research Network—Jönköping (ARN‐J), School of Health and Welfare, Jönköping University Jönköping Sweden; ^6^ Aging Research Center, Department of Neurobiology, Care science and Society Karolinska Institutet Stockholm Sweden

**Keywords:** home care service, home care staff, job strain, older person, psychosocial working conditions, satisfaction with care

## Abstract

Older persons in Sweden are increasingly encouraged to continue living at home and, if necessary, be supported by home care services (HCS). Studies have examined whether the work environment of staff has an impact on the experiences and well‐being of older persons in residential care facilities, but few have examined such associations in HCS. This study examined associations between home care staff's perceptions of their psychosocial work environment and satisfaction with care among older people receiving HCS. The setting was 16 HCS work units. Two surveys were conducted, one on psychosocial working conditions of staff, one on satisfaction of older persons receiving HCS. For each work unit, data on individual satisfaction were matched to average values concerning psychosocial work conditions. Outcomes analysed with linear regressions were overall satisfaction and indices regarding assessment of performance of services, contact with staff and sense of security. The index for treatment by staff was analysed with ordered logistic regressions. Cluster correlated‐standard error clustering on work units was used. Results showed that good working conditions were important for satisfaction with care, specifically overall satisfaction, treatment by staff and sense of security. The most important psychosocial work factors were work group climate, sense of mastery, job control, overall job strain, frustrated empathy, balancing competing needs, balancing emotional involvement and lack of recognition. Receiving more HCS hours was associated with stronger relationships between working conditions and satisfaction with care, especially with overall satisfaction and treatment by staff as outcomes. Managers and policymakers for home care need to acknowledge that the working conditions of home care staff are crucial for the satisfaction of older persons receiving HCS, particularly those receiving many HCS hours. Psychosocial work factors together with job strain factors are areas to focus on in order to improve working conditions for staff and outcomes for older persons.

**What is known about this topic**

- More older persons with increasingly complex needs are living at home with support from home care services.
- The working conditions of home care staff are reported to be demanding and staff have reported high levels of job strain.

**What this paper adds**

- Factors in the work environment, such as work group climate, sense of mastery and job control, are associated with satisfaction with care among older persons receiving home care services.
- A high level of job strain among staff is associated with less satisfaction with care among older persons receiving home care services.
- Improvement in the work environment will not only be beneficial for staff, but better outcomes for older persons are also expected.

## 1 INTRODUCTION

In accordance with the Swedish ‘ageing in place’ policy, older persons in Sweden are increasingly encouraged to continue living at home and, if necessary, be supported by home care services (HCS) and family caregivers (The Swedish National Board of Health and Welfare, 2016, 2017). However, the decline in residential care since the turn of the century has not seen a corresponding increase in the availability of home care (Ulmanen & Szebehely, 2015). Provision of HCS is decided on a need basis, which is assessed by social workers in the municipalities (Wolmesjö & Staaf, 2014). The support offered by HCS to persons in need includes personal care and domestic tasks such as cooking and shopping, with a varying range of service hours depending on the needs of the older person (Sandberg et al., 2019). Dignity and respect are the core values in the Swedish national guidelines concerning the care of older persons. A recent longitudinal study indicated that satisfaction with HCS in relation to dignity and respect declined over time (2016–2018) among persons both with and without dementia (Hammar et al., 2021). Recounting their experiences of being cared for at home, older persons described it as becoming a guest in their own home, that their privacy was compromised, and that they had very little influence over their care (Jarling et al., 2018). Thus, there is a need to enhance the satisfaction with HCS among older persons since an increasing number of older people will be dependent on this service.

Home care staff meet a heterogenous group of older persons in their work. The older persons can have different medical diagnoses and therefore vary in terms of complexity of care needs, cognitive ability, functional limitations and other factors (Sandberg et al., 2019). Such variations require the staff to adapt their care provision accordingly in order to provide person‐centred care. For example, the core process of providing person‐centred care for persons with dementia is enacting and re‐enacting familiarity (Hedman et al., 2021). Older persons receiving home care also emphasise the importance of continuity and familiarity with home care staff for their own sense of stability in the care delivered (Olsen et al., 2020). For the home care staff, feelings of inadequacy in the provision of care can lead to perceived job strain (Sandberg et al., 2018). Other significant factors contributing to job strain are reported to be a stagnant organisational climate, lack of recognition of their work and not having Swedish as their first language, leading to difficulties in understanding between home care staff and the older persons. A review of the literature reports lower job stability, pay, working hours and fringe benefits among home care aides compared to those working in hospitals and residential care facilities (RCF) (Hewko et al., 2015).

Studies have examined whether the work environment of staff has an impact on the experiences and well‐being of the older persons they care for. For example, Swedish studies in RCF have shown that a positive work climate, in which staff experience job satisfaction and support in their work, has positive associations with the older persons' ratings of the quality of care and how well they thrive in the RCF (Edvardsson et al., 2008; Lundgren et al., 2019, 2020). A Canadian study has also reported positive associations between a supportive work context and the clinical outcomes of older residents living in RCF (Estabrooks et al., 2015). To our knowledge, few studies have examined whether there are also associations between older persons' perceptions of care and staff's perceptions about their work situation in HCS. A recent study conducted in RCF and home care in one Swedish municipality identified stronger associations between staff work environment and the satisfaction of older people with their care in the RCF settings than in the home care settings (Lundgren et al., 2020). In the study by Lundgren et al. (2020), the researchers did not have information about the number of HCS hours that the older persons had been granted and could not control for this. Since home care for older people will increase and there are calls for enhancing the quality of care and satisfaction with care, there is a need to explore if there are significant associations between older persons' and staff's perceptions in a larger sample including several municipalities. If associations between staff's work environment and older persons' satisfaction with home care are found, interventions could be tailored to modify these aspects in a positive and beneficial direction. Thus, the current study examined the association between home care staff's perceptions of their psychosocial work environment and satisfaction with care among older people receiving HCS.

## 2 MATERIAL AND METHODS

### 2.1 Study design

This cross‐sectional study combines data from three different data sources to capture the perceptions from older persons receiving HCS and home care staff. Every year, since 2012, the Swedish National Board of Health and Welfare (NBHW) invites all persons aged 65 years and older who have been granted HCS to participate in a national User Satisfaction Survey (USS) concerning their experiences with HCS (The National Board of Health and Welfare, 2012). The USS data from 2018 were combined with data from a national HCS register over support that has been granted; this register is managed by NBHW. The third source of data was a survey conducted among home care staff concerning their psychosocial working conditions at agencies providing HCS to older persons during the same time period as the USS data were collected.

### 2.2 Setting and samples

The geographical setting for this study was Stockholm County, Sweden. All 26 municipalities in the county were invited to take part in the study and five agreed to participate. Within each municipality, HCS are provided by public or private home care agencies. The selected agencies had to meet the inclusion criteria of (1) having at least 30 employees who had been employed for at least 3 months and had a contract corresponding to ≥50% employment (20 h/week) and (2) providing HCS to at least 30 persons. The criteria were met by five agencies, two private and three public, which included 16 work units in total.

#### 2.2.1 Older person

The national USS was sent to all persons aged 65 years and older receiving HCS. The sample consisted of older persons receiving HCS from the 16 work units. Of these, 1239 (60%) participated. Proxy interviews were excluded, resulting in an analytical sample of 723 persons.

#### 2.2.2 Home care staff

The inclusion criteria were working in the homes of older persons and having a permanent employment contract or having had a temporary position for at least 3 months. Four hundred and sixty‐seven staff members met the criteria in the 16 included work units. Of these, 219 (47%) participated.

### 2.3 Data

*The USS survey* of older persons includes 25 questions about overall satisfaction with care, treatment by staff, performance of services, contact with staff and sense of security (The National Board of Health and Welfare, 2012f). The USS survey data were supplemented with individually matched data from the HCS register concerning number of HCS hours granted and type of support provided for each person.

The staff survey consisted of two self‐reported instruments to assess psychosocial working conditions, the *Strain in Dementia Care Scale* (SDCS) and the QPSNordic 34+ (QPS). SDCS addresses the perceived level of job strain among care staff (Edberg et al., 2008, 2015). The QPS measures other psychosocial aspects of the work environment and is developed from organisational theories to investigate the relationship between work, health and productivity (Dallner et al., 2000; Lindström et al., 2000; Wännström et al., 2009).

### 2.4 Dependent variables

The USS includes one question on overall satisfaction with care, 10 additional questions on satisfaction with care and one general question on treatment by staff. The response alternatives were: yes, always/often/sometimes/seldom/never, which were coded as either 4–0 or 0–4 so that positive answers were given a higher value. Item non‐response was between 4 and 36 for each of the questions. Multiple imputations based on multivariate normal regressions were conducted on the questions concerning satisfaction with care, but not for the question concerning overall satisfaction. The general question on treatment by staff was followed by nine yes/no questions concerning specific negative aspects of treatment by staff; yes is coded 0, no is coded 1 (no is the positive answer).

Five dependent variables were constructed using the USS. These were *overall satisfaction, treatment by staff, performance of services, contact with staff* and *sense of security*. *Overall satisfaction* was a single‐item variable. For *treatment by staff*, a summed index was created from the general question and the nine specific questions, ranging from 5 to 13 (theoretically ranging from 0 to 13), mean 12.5. The 10 additional questions were included in a principal component analysis (PCA) with varimax rotation (Jolliffe, 2002; StataCorp., 2021a). A three‐factor solution was suitable. The result is presented in Table S1. Based on the three factors obtained from the PCA, three indices, *performance of services, contact with staff, sense of security*, were created by adding the answers and dividing by the number of questions. Zero‐skewness log transformation and z‐transformation were used on the variables, except for *treatment by staff* where 71% had a score of 13, corresponding to best treatment. All variables were skewed. Zero‐skewness log transformation was used to obtain variables without skewness, suitable for linear regression (Box & Cox, 1964; StataCorp., 2021b). Z‐transformations involve the original variables being subtracted by the mean and divided by the standard deviation to obtain variables with a mean of zero and a standard deviation of 1.0 (Gujarati, 1988). This makes the results for different variables more comparable.

### 2.5 Independent variables

The *SDCS* consists of 27 statements concerning the staff's work situation. For each statement, two aspects were investigated: (1) how frequently the situation occurs and (2) how much stress each situation generates. Both aspects are measured on a four‐point Likert scale: 1 = never/no stress to 4 = very often/high stress. The level of perceived job strain is calculated by multiplying the response of frequency and stress for each statement, which creates an output between 1 and 16. A higher number indicates a higher perception of job strain (Edberg et al., 2015). The *QPS* consists of 37 questions with answers ranging from very seldom or never to very often or always (coded 1–5) (Dallner et al., 2000; Lindström et al., 2000; Wännström et al., 2009). In the sample of 219 staff, item non‐response was 15–24 for the variables based on the SDCS questions and 2–6 for the QPS questions. Multiple imputations were performed separately for the SDCS variables and the QPS variables.

The 27 SDCS statements were categorised into five factors: *frustrated empathy* (7 items), *difficulty understanding and interpreting* (7 items), *balancing competing needs* (5 items), *balancing emotional involvement* (4 items) and *lack of recognition* (4 items) (Edberg et al., 2015). Each factor is based on the average of the included items. In addition, all 27 statements were used as a *total job strain* variable. The statements are presented in Table S2. This method of coding has been used previously by our research group (Fallahpour et al., 2020; Sandberg et al., 2018).

There is no pre‐defined solution for creating indices from the *QPS* questions. We have included them in a PCA with varimax rotation, obtaining six factors. Six summed indices based on the average of the included items were constructed based on the result of the PCA (Table S3). These were *support from manager, group work climate, sense of mastery, job control, social work environment* and *positive challenges*. One overall variable was constructed from the summed index of all QPS questions, *QPS total*. For the QPS index, high means good working conditions. *QPS total* and *total job strain* were z‐transformed.

Background information regarding the staff included age, sex, length of employment, having Swedish as mother tongue/not, being care assistants/other occupation, permanent employment/not, working full time/not, education (lower/upper secondary/university) and care education/not. Furthermore, staff self‐reported health was assessed using a summed index based on questions about depressive symptoms, sleep disturbances, feeling worried and fatigue (Engström et al., 2006). Averages and proportions were calculated for all these variables within each work unit. The averages and proportions from each work unit have been merged with the information from the persons receiving care from the staff who were working in that unit.

Preliminary analyses showed that the work unit characteristic regarding the proportion of participants having a *university education* had the strongest association with the dependent variables concerning satisfaction with care. This variable is therefore included as a separate control variable. Due to the high correlations between the other work unit characteristics and average psychosocial working conditions (Table S4), a single factor was created with a one‐factor solution from a PCA with varimax rotation based on work unit characteristics (except for working conditions and university education). This factor and the proportion having a university education were used as control variables in the last model of the regression analyses.

Control variables for the older persons were sex, age, cohabiting/not and health. None had missing information concerning sex and age, and 24 had missing information concerning cohabitation/not. A new variable was created with three categories: cohabiting/not/missing. Health was measured as global self‐rated health (nine missing), symptoms of anxiety (12 missing) and mobility (14 missing). Multiple imputations based on multivariate normal regressions were used. A summed index was created from the resulting health variables.

Information about the number of HCS hours granted for each older person was obtained from the HCS register.

### 2.6 Data analysis

All analyses were transformed with STATA version 16. P‐values for the descriptive statistics were based on χ^2^ tests or binary logistic regressions with overall satisfaction low/high as outcome. The dichotomisation into low/high overall satisfaction was based on median split.

Due to the skewed distribution, *treatment by staff* was analysed with ordered logistic regression. After zero skewness log transformation, the single item question on overall satisfaction and the other three indices were non‐skewed and analysed with linear regressions. There might be a clustering of good or bad opinions about the care the older persons receive depending on the unit, which might lead to erroneously low standard errors. Cluster‐correlated standard errors, based on the 16 work units, have been used to control for this (White Jr., 1980).

The results are presented as β‐coefficients and p‐values. The β‐coefficients for treatment by staff are from ordered logistic regressions and cannot be compared to the β‐coefficients for other outcomes. The analyses were made in two models. *Model 1*: No controls but includes interaction between psychosocial working conditions and HCS hours given linear representation. HCS hours were centred at Q3 (the 75th percentile, 146 h/month). Coefficients for the interactions are also presented. The *interactions* presented in Model 1 show if the association between independent variables regarding psychosocial work environment had a different association with satisfaction with care depending on the number of HCS hours. Since the number of hours was centred at 146 h, the results for the *main effect* were the estimated association among people receiving 146 HCS hours. *Model 2*: Interactions with *p* < 0.050 in Model 1 are included in Model 2. In addition, this was controlled for older person and work unit characteristics as described earlier.

### 2.7 Ethical considerations

This study was approved by the Regional Ethical Committee, Stockholm, Sweden (Dnr: 2018/449–31/5). Ethical information was provided to all participants (older persons and staff) before they began filling in the questionnaire. By handing in their responses, the participants gave their informed consent.

## 3 RESULTS

### 3.1 Description of samples

The characteristics for the two samples (723 older persons and 219 home care staff) are presented in Table 1 and Table 2. In the group of older persons, two‐thirds were women and the mean age was 85 years (range 66–104). Each person received, on average, 28 HCS hours/month (range 1–177 hours). Among home care staff, the majority were women (80%) and the mean age was 48 years. The mean length of HCS employment was 13 years and 57% had Swedish as their first language.

**TABLE 1:** Descriptive statistics for the older persons receiving home care services

Variable described^a^		Overall satisfaction (single item)		
	All	Low	High	*p*‐value^b^
Number of observations	723	370	353	
Dependent variables, the older person's satisfaction with care (log variables are z‐transformed)				
Treatment by staff^c^	12.5	12.2	12.9	**<0.001**
Performance of services	3.0	2.7	3.2	**<0.001**
Log performance of services	0	−0.31	0.32	**<0.001**
Contact with staff	2.9	2.5	3.3	**<0.001**
Log contact with staff	0	−0.46	0.48	**<0.001**
Sense of security	3.2	2.8	3.6	**<0.001**
Log sense of security	0	−0.63	0.66	**<0.001**
Overall satisfaction (single item)	3.3	2.7	4.0	**<0.001**
Log overall satisfaction (single item)	0	−0.93	0.97	**<0.001**
Other characteristics of the older persons				
Self‐reported questionnaire (vs. mixed^d^)	90%	89%	92%	0.243
Woman	67%	69%	66%	0.403
Age, mean (range 66–104)	85	85	84	0.215
Service hours/month, (range 1–177)	28	30	25	**0.039**
Cohabiting, Yes	19%	18%	19%	
No	78%	79%	77%	
Missing	3%	3%	4%	0.874
Health problems (range 0–10.7)	4.0	4.4	3.7	**<0.001**

^a^ Mean values, except when % are indicated after the variable.

^b^ *p*‐value for difference between low and high. Based on χ^2^ tests for discrete variables and binary logistic regressions for ordinal variables.

^c^ Theoretical range 0–13, observed range 5–13.

^d^ Another person assisted the older person with completion of the survey.

Bold value indicates *p* < 0.05.

**TABLE 2:** Descriptive statistics for the home care staff (*n* = 219)

Psychosocial working conditions, SDCS (high is high level of job strain) (theoretical range 1–16)			
	Min	Max	Mean
Frustrated empathy	3.8	5.7	4.7
Difficulties understanding and interpreting	1.9	3.9	3.1
Balancing competing needs	3.6	6.8	5.2
Balancing emotional involvement	2.5	6.8	4.6
Lack of recognition	4.3	8.0	5.3
Total job strain (z‐transformed)	−0.58	0.82	0
QPS (high is good) (theoretical range 1–5)			
Support from manager	2.2	3.9	2.9
Work group climate	2.7	4.2	3.5
Sense of mastery	2.4	4.0	3.4
Job control	1.0	3.1	2.4
Social environment at work	2.8	4.2	3.5
Positive challenges	3.5	4.3	4.0
QPS total (z‐transformed)	−0.72	0.89	0
Other staff characteristics			
University education			21%
Age (min/max/mean)	18	67	48
Woman			80%
Swedish as mother tongue			57%
Nursing assistant			36%
Permanent position			92%
Fulltime position			72%
Length of employment (years)	0	40	13
Education < upper secondary			17%
Care education			85%

*Note*: Based on total sample of staff included in the study.

### 3.2 Associations between psychosocial work environment and satisfaction with care

Table 3 shows the results of the regression analysis between psychosocial working conditions and satisfaction with care. The results are presented as β‐coefficients and p‐values. Significant results (*p* < 0.05) are marked in bold. The general pattern is that better psychosocial working conditions (lower values for job strain, higher values on QPS) were often significantly associated with the outcomes: *overall satisfaction* (column 1), *treatment by staff* (column 2) and *sense of security* (column 5), but not often associated with *performance of services* (column 3) and *contact with staff* (column 4). Among the independent variables, the job strain factors *frustrated empathy*, *balancing competing needs, balancing emotional involvement*, *lack of recognition* and *total job strain* and the QPS variables (see lower half of Table 3) *support from manager*, *work group climate*, *sense of mastery*, *job control* and *QPS total* demonstrated associations with the dependent variables *overall satisfaction*, *treatment by staff* and *sense of security*.

**TABLE 3:** The associations between psychosocial working conditions and the older person's satisfaction with home care services

	Dependent variables				
	Overall satisfaction (single item)	Treatment by staff	Performance of services	Contact with staff	Sense of security
Independent variables					
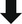	β (*p*‐value)^a^	β (*p*‐value)^b^	β (*p*‐value)^a^	β (*p*‐value)^a^	β (*p*‐value)^a^
Job strain variables from SDCS (high value indicates high level of job strain)					
Frustrated empathy					
Model 1	**−0.874 (0.002)**	**−1.503 (<0.001)**	−0.210 (0.327)	−0.281 (0.347)	**−0.745 (0.001)**
Interaction^c^	**−0.070 (0.001)**	**−0.094 (<0.001)**	−0.020 (0.265)	−0.012 (0.558)	**−0.051 (0.006)**
Model 2	**−0.936 (0.001)**	**−1.781 (0.001)**	−0.321 (0.288)	−0.085 (0.387)	−0.120 (0.239)
Difficulties understanding and interpreting					
Model 1	0.105 (0.809)	0.318 (0.783)	−0.093 (0.767)	0.885 (0.079)	0.570 (0.253)
Interaction^c^	0.015 (0.629)	0.042 (0.650)	0.003 (0.906)	0.068 (0.061)	0.053 (0.146)
Model 2	−0.110 (0.285)	−0.194 (0.491)	**−0.182 (0.017)**	−0.019 (0.904)	−0.115 (0.228)
Balancing competing needs					
Model 1	**−0.531 (0.017)**	**−0.373 (0.012)**	−0.267 (0.117)	−0.019 (0.927)	**−0.360 (0.030)**
Interaction^c^	**−0.033 (0.040)**	−0.017 (0.083)	−0.016 (0.350)	0.005 (0.656)	−0.018 (0.100)
Model 2	**−0.516 (0.004)**	**−0.440 (<0.001)**	−0.072 (0.301)	−0.033 (0.524)	−0.099 (0.101)
Balancing emotional involvement					
Model 1	−0.371 (0.064)	**−1.338 (0.001)**	−0.176 (0.240)	0.015 (0.937)	**−0.443 (0.017)**
Interaction^c^	**−0.033 (0.033)**	**−0.077 (0.008)**	−0.011 (0.483)	0.002 (0.899)	**0.029 (0.036)**
Model 2	**−0.528 (0.007)**	**−1.576 (<0.001)**	−0.085 (0.228)	−0.043 (0.499)	**−0.515 (0.004)**
Lack of recognition					
Model 1	**−0.454 (0.006)**	**−0.751 (0.009)**	−0.033 (0.793)	0.020 (0.898)	**−0.320 (0.034)**
Interaction^c^	**−0.032 (0.010)**	−0.040 (0.051)	0.002 (0.892)	0.009 (0.389)	−0.017 (0.139)
Model 2	**−0.461 (0.009)**	**−0.289 (0.010)**	−0.051 (0.371)	−0.056 (0.308)	−0.081 (0.132)
Total job strain					
Model 1	**−0.412 (0.004)**	**−0.777 (<0.001)**	−0.116 (0.312)	0.011 (0.925)	**−0.305 (0.007)**
Interaction^c^	**−0.029 (0.009)**	**−0.041 (0.008)**	−0.006 (0.578)	0.005 (0.482)	**−0.017 (0.039)**
Model 2	**−0.438 (0.002)**	**−0.859 (<0.001)**	−0.050 (0.252)	−0.037 (0.432)	**−0.307 (0.005)**
QPS variables (high value indicates positive work conditions)					
Support from manager					
Model 1	**0.748 (0.005)**	**1.074 (0.007)**	0.087 (0.548)	0.247 (0.399)	0.448 (0.134)
Interaction^c^	**0.065 (0.001)**	**0.073 (0.024)**	−0.010 (0.498)	0.009 (0.641)	0.030 (0.188)
Model 2	**0.645 (0.025)**	1.174 (0.101)	**0.233 (0.012)**	0.025 (0.853)	0.002 (0.991)
Work group climate					
Model 1	**1.193 (<0.001)**	**1.747 (<0.001)**	0.259 (0.077)	**0.887 (0.002)**	**0.908 (0.004)**
Interaction^c^	**0.079 (0.001)**	**0.067 (0.033)**	0.010 (0.538)	0.034 (0.154)	0.043 (0.083)
Model 2	**1.180 (<0.001)**	**2.111 (<0.001)**	0.146 (0.097)	**0.418 (0.014)**	**0.399 (0.040)**
Sense of mastery					
Model 1	**1.627 (<0.001)**	**2.430 (<0.001)**	0.038 (0.894)	0.412 (0.347)	**1.187 (<0.001)**
Interaction^c^	**0.121 (<0.001)**	**0.144 (0.001)**	−0.016 (0.503)	0.012 (0.655)	**0.072 (0.007)**
Model 2	**1.759 (0.001)**	**2.977 (<0.001)**	**0.412 (0.013)**	0.386 (0.074)	**1.215 (0.014)**
Job control					
Model 1	**1.072 (<0.001)**	**1.717 (<0.001)**	−0.034 (0.861)	0.332 (0.274)	**0.834 (<0.001)**
Interaction^c^	**0.081 (<0.001)**	**0.090 (<0.001)**	−0.015 (0.374)	0.018 (0.332)	**0.052 (0.007)**
Model 2	**1.141 (0.002)**	**2.886 (<0.001)**	0.251 (0.121)	0.058 (0.783)	**0.822 (0.043)**
Social environment at work					
Model 1	0.745 (0.219)	1.433 (0.075)	−0.063 (0.816)	0.141 (0.675)	0.480 (0.422)
Interaction^c^	0.082 (0.095)	**0.149 (0.005)**	−0.005 (0.870)	0.017 (0.506)	0.049 (0.326)
Model 2	**−0.220 (0.047)**	1.207 (0.225)	−0.021 (0.899)	−0.047 (0.583)	−0.119 (0.311)
Positive challenges					
Model 1	1.497 (0.062)	1.315 (0.319)	−0.225 (0.642)	0.708 (0.267)	0.661 (0.352)
Interaction^c^	0.107 (0.057)	0.050 (0.496)	−0.017 (0.371)	0.040 (0.298)	0.018 (0.706)
Model 2	0.066 (0.757)	0.726 (0.324)	−0.078 (0.653)	−0.004 (0.984)	0.300 (0.097)
QPS total					
Model 1	**0.417 (<0.001)**	**0.612 (<0.001)**	0.013 (0.860)	0.161 (0.152)	**0.276 (0.011)**
Interaction^c^	**0.033 (<0.001)**	**0.034 (0.024)**	−0.004 (0.463)	0.006 (0.374)	0.016 (0.115)
Model 2	**0.397 (<0.001)**	**0.742 (0.006)**	**0.082 (0.004)**	0.058 (0.397)	0.077 (0.208)

*Note*: (1) Linear regressions and (2) ordered logistic regressions with cluster‐correlated standard errors with work unit as cluster (*n* = 16). Psychosocial working conditions are the average for each of the work units given linear representation. Note that β‐coefficients for treatment by staff are based on ordered logistic regressions and not comparable with β‐coefficients for other dependent variables that were analysed with linear regressions.

*Note*: Bold indicates *p* < 0.05.

*Note*: Model 1: No controls but including interaction between psychosocial working conditions and hours of home care service given linear representation. Home care service hours were centred at Q3 (the 75th percentile, 146 h). Coefficient for the interaction is also presented.

*Note*: Model 2: Interaction with *p* < 0.050 in model 1 were included in model 2. Additionally controlling for older person's characteristics (sex, cohabiting/not, age and health given linear representation) and work group characteristics (university education and factor from principal component analyses with a one factor solution, including average age, length of employment, health, woman, Swedish as mother tongue, nursing assistant, permanent employment, working full time, education lower than upper secondary and care education).

^a^ β and *p*‐value in linear regressions.

^b^ β and *p*‐value in ordered logistic regressions.

^c^ Coefficient and *p*‐values for interaction between psychosocial working conditions and home care service hours given linear representation.

The job strain variable *difficulties understanding* was not significantly associated with any dependent variables (except for a significant association with *performance of services* in Model 2). The QPS variables *social work environment* (except for a negative association with *overall satisfaction*) and *positive challenges* did not have any significant associations with any dependent variables.

The interactions presented in Table 3 indicate whether the association between the staff's psychosocial working conditions and older persons' satisfaction with care differs with number of HCS hours. For job strain variables, a negative coefficient for the interaction indicates that the associations are stronger with a larger number of HCS hours. For the QPS variables, positive coefficients indicate a stronger association with more hours. In all instances except one (the association between *balancing emotional involvement* and *sense of security*), significant interactions (marked in bold) show that the association between psychosocial working conditions and satisfaction with home care is stronger with more hours of home care. For *emotional involvement* and *sense of security*, significant interactions indicated that the associations are stronger among people receiving more HCS hours.

In general, the results showed that the number of HCS hours is important for the association between the job strain factors *frustrated empathy*, *balancing emotional involvement* and *total job strain* and older people's assessment of *overall satisfaction*, *treatment by staff* and *sense of security*. In other words, if staff assess having less *frustrated empathy*, then *balancing competing needs*, *balancing emotional involvement* and *lack of recognition* become more important for the older persons' assessments of *overall satisfaction* the more HCS hours the older persons are granted. In line with the findings for job strain factors, the number of HCS hours is also important for the association between the QPS variables *support from manager*, *group climate*, *job control* and *QPS total* and the outcomes *overall satisfaction* and *treatment by staff*. The number of HCS hours is also important for the association between *job control* and *sense of security*.

## 4 DISCUSSION

This study shows strong associations between older persons' level of satisfaction with their care and the level of satisfaction with working conditions reported by staff. In particular, the older persons' ratings of *overall satisfaction*, *staff treatment* and *sense of security* were positively associated with staff's psychosocial working conditions and a low rating of job strain. Since the research regarding associations between older persons' satisfaction with their care and staff's perceptions about their work organisation in the home care setting is very limited, we will to some extent discuss our findings in relation to the literature from RCF settings. In line with our findings, a systematic review of 25 articles in the field of RCF reported positive relationships between resident satisfaction and staff job satisfaction (Li et al., 2021).

The analysis between *overall satisfaction, staff treatment* and *sense of security* and the independent variables revealed a pattern whereby the five independent variables *frustrated empathy*, *balancing emotional involvement*, *work group climate*, *sense of mastery* and *job control* were consistently associated with the older persons' ratings. In previous home care studies, staff reported high job demands with few possibilities for having some level of control over their daily work situation (Grasmo et al., 2021; Möckli et al., 2020). Other studies have reported high levels of job demands being strongly associated with low job satisfaction (Gleason & Miller, 2021) and also high job strain (Orrung Wallin et al., 2015; Sandberg et al., 2018). In this study, *sense of mastery* is a concept that is the opposite of job demands meaning that those who perceive a sense of mastery in their work are able to manage their job demands. It is vital that the home care staff members can master their job, for example being sufficiently skilled and experienced to manage the work tasks, since they often work without a colleague in the older person's home. Furthermore, in order to master their work, the staff need support. This is indicated by our findings that *work group climate* seems to be an important factor for delivering care that is assessed as satisfactory by older persons receiving home care. Previous research in RCF has reported that leadership support is essential (Haunch et al., 2021). In our findings, *support from managers* was strongly associated with the older person ratings of *overall satisfaction*, *treatment by staff*, but not with *sense of security*, while the staff's ratings of *work group climate* were strongly associated with four out of five outcomes. Home care managers often supervise a large group of staff (on average 42 staff members) and being able to have a supportive relationship with each staff member is difficult (The Swedish National Board of Health and Welfare, 2021). In light of this, the work group is therefore the closest potential source of support for the staff member in problematic situations. This issue has been identified in Sweden as an important aspect that needs to be changed so that the managers have fewer staff members to supervise (The Swedish National Board of Health and Welfare, 2021).

Regarding factors related to job strain, *frustrated empathy* and *balancing emotional involvement* were associated with satisfaction with care reported by the older persons. The SDCS items assess strain in relation to different care situations involving staff and the older person or family member. As described above, the home care staff work predominantly by themselves in the older person's home. They need to have the skills and experience to handle many different tasks, from preparing meals, cleaning and laundry to assessing healthcare needs, administering medications and arranging contact with healthcare staff for older persons with complex care needs and functional limitations (Craftman et al., 2018; Sandberg et al., 2018). The staff are often not well educated for these different tasks and access to continuing education is very limited. Staff working in home care have reported higher job strain than staff working in RCF. Home care staff working with persons with dementia have reported a significantly higher level of job strain regarding *frustrated empathy, balancing competing needs* and *lack of recognition* compared with other home care staff (Orrung Wallin et al., 2015; Sandberg et al., 2018). Furthermore, organisational factors, such as job demands and organisational culture, have been shown to be associated with job strain, indicating that the psychosocial work conditions will influence staff's perceived job strain in RCF (Orrung Wallin et al., 2015).

The associations were stronger the more HCS hours the older persons had been granted. More HCS hours implies that these persons have more and often complex care needs and thereby are more dependent on the care that is given. A larger number of staff members are involved and there might therefore be a lack of continuity of staff. These older persons are more exposed to sensitive situations, due to poor health and requiring intimate care, factors that make the older person more perceptive to how the care is provided (Ernsth Bravell et al., 2021). A previous study has also reported that a greater number of HCS hours was related to dissatisfaction among older persons with home care services compared with persons granted less HCS hours (Hammar et al., 2021).

The older persons' ratings regarding *performance of services* and *contact with staff* were not consistently associated with any of the variables regarding staff‐reported job strain or psychosocial working conditions. One explanation for this finding could be that *performance of services* and *contact with staff* include items that are more task‐oriented and do not capture the relationships with staff. However, previous research has shown that if leaders give support, show interest in the staff's well‐being, and focus on empowering leadership, the staff's assessment of their psychosocial work environment will improve (Lundgren et al., 2019). Furthermore, the independent variables *social environment at work, positive challenges* and *difficulties understanding and interpreting* were not consistently associated with any of the five outcome measures reported by older persons. The latter finding was of interest since some previous studies have reported that there are problems with staff having difficulty understanding the older persons and their family members, particularly staff members who do not have Swedish as their first language (Rosendahl et al., 2016; Söderman & Rosendahl, 2016). Our results do not support an association between staff not having Swedish as their native language and satisfaction with home care among older persons.

The findings from this study can be used to develop and evaluate different interventions with the aim of improving satisfaction with care through modifying psychosocial work conditions, which had the strongest associations with older persons' ratings of satisfaction with care. For example, *work group climate* was associated with four of the five outcome measures; it was, in particular, strongly associated with *overall satisfaction* and *treatment by staff* indicating that increased ratings of work group climate might enhance the ratings by the older persons. The items in the index *work group climate* contain aspects concerning the climate in the group being encouraging, supportive and relaxing, and also that the group is able to solve problems. Managers in home care can therefore support staff members in finding ways to solve problems, and facilitate group meetings during the working day, so that staff members will have time to support each other. Regarding job strain, a pilot study that evaluated an educational model focused on implementing person‐centred care in the home care of older persons with dementia found reduced job strain among the staff who participated in the education (Fallahpour et al., 2020). In RCF, satisfaction among residents has enhanced by arranging training programs for staff, quality of daily interaction with staff and the overall contribution of staff in improving residents' experiences (Li et al., 2021).

### 4.1 Strengths and limitations

This study used a cross‐sectional design, and the participants were selected from five municipalities in Stockholm County, Sweden. The inclusion criteria of the sample (agencies), and the characteristics of the samples may affect the generalisability of the results; we do not know whether the HCS in other municipalities in Sweden share these characteristics. Two of the questionnaires used in this study (SDCS & USS) were developed in Sweden, and development of the QPS questionnaire was the result of a collaboration between researchers from four Nordic countries (Finland, Norway, Sweden & Denmark). The questionnaires have shown adequate validity and reliability. QPSNordic 34+ is a short version of the QPSNordic and contains questions from the scales of the QPSNordic, but each scale is only represented by one or two questions; a PCA was therefore performed. The response rates for the questionnaires were relatively low (staff [SDCS & QPS], 47% and older persons [USS], 60%). We did not include staff who were hourly employees, and we have no information about how many staff had this type of employment contract, which is a weakness. Selection bias due to non‐response may have affected the results. The study used several different measures of satisfaction with care among older persons and the staff's psychosocial working conditions, thus providing a clear picture of the associations between home care staff's psychosocial working conditions and satisfaction with care among older persons. A strength is that we have used cluster‐correlated standard errors, clustered for work groups, to control for the possible aggregation of similar answers within the work units. Future research studies should include a larger number of home care agencies from more municipalities in different Swedish counties in order to obtain a representative sample of home care staff and older persons.

### 4.2 Conclusions

Managers of, and policymakers for, home care need to acknowledge that the working conditions of home care staff are important for the satisfaction with the care experienced by the older persons receiving this service, particularly for persons granted a high number of HCS hours. The psychosocial work conditions *job control*, *sense of mastery* and *work group climate* together with job strain factors *frustrated empathy* and *balancing emotional involvement* are strongly associated with outcomes regarding *overall satisfaction*, *treatment by staff* and *sense of security*; these are areas to focus on in order to improve the working conditions of home care staff.

## AUTHOR CONTRIBUTIONS

Study design: AMB, DL, ZNK and IK. Data collection: AMB was responsible for data withdrawal from the NBHW and the data collection in the home care agencies. Analysis: AMB, DL and IK conducted the analysis. AMB, DL, ZNK and IK reviewed and commented on the analysis. Drafting of the article: AMB drafted the article, and all authors reviewed and commented on drafts of the manuscript. All authors read and approved the final version of the manuscript.

## FUNDING INFORMATION

This study was funded by grants from The Swedish Research Council for Health, Working Life and Welfare (FORTE) for the research program *Future Care for Older Adults in Home Care and Care Home* DNR. 2016–07089. The funding body had no role in the design of the study, data collection, analysis, interpretation of data or in writing the manuscript.

## CONFLICT OF INTEREST

The authors declare that they have no competing interests.

## Supporting information


Table S1

Table S2

Table S3

**Table S4**.

## Data Availability

Data available on request from the first author.
