# Supplementary material for: Factors in the psychosocial work environment of staff are associated with satisfaction with care among older persons receiving home care services
Source: Health Soc Care Community. 2022 Sep 26;30(6):e6080–90. doi: 10.1111/hsc.14045 (PMC10087462; doi:10.1111/hsc.14045)
Supplement: Supplementary file 1 — Table S1 [file HSC-30-e6080-s004.docx]

Supplementary Table 1. Results from principal component analyses with varimax rotation of questions from the user satisfaction survey

|  | Component | | |  |
| --- | --- | --- | --- | --- |
|  | 1 | 2 | 3 |  |
| Item/question | Factor loadings | | | |
| **Performance of services** |  | | |  |
| Consideration by staff of views and wishes | .732 |  |  |  |
| Performance of services by staff | .798 |  |  |  |
| Staff having enough time | .491 |  |  |  |
| Trust in staff | .700 |  |  |  |
| Collaboration with staff | .835 |  |  |  |
| **Contact with staff** |  |  |  |  |
| Possibility to decide what time staff arrive |  | .842 |  |  |
| Staff arrive as scheduled |  | .695 |  |  |
| Information from staff |  | .757 |  |  |
| **Sense of security** |  |  |  |  |
| Feeling of safety |  |  | .729 |  |
| Easy to get in contact with staff |  |  | .761 |  |
