# Supplementary material for: Factors in the psychosocial work environment of staff are associated with satisfaction with care among older persons receiving home care services
Source: Health Soc Care Community. 2022 Sep 26;30(6):e6080–90. doi: 10.1111/hsc.14045 (PMC10087462; doi:10.1111/hsc.14045)
Supplement: Supplementary file 2 — Table S2 [file HSC-30-e6080-s002.docx]

Supplementary Table 2. Presentation of Strain in Dementia Care Scale factors and items

| **Strain in Dementia Care Scale** |
| --- |
| **Factor 1 Frustrated empathy** |
| I see other staff behaving towards an older person in a way that shows they do not understand the effects of dementia |
| I see that an older person is suffering |
| Older persons do not receive the care I feel they are entitled to |
| I see the families of older persons suffering |
| I see older persons being mistreated by their family |
| I see other staff treating the older persons badly |
| Other staff try to change what I have done for an older person |
| **Factor 2 Difficulties understanding and interpreting** |
| I have difficulties understanding what older persons are thinking |
| I have difficulties understanding what older persons are trying to communicate |
| I have difficulties understanding the needs of the older persons |
| I find it difficult to know what is best for older persons |
| I worry I might upset or hurt an older person because I don’t understand their needs |
| I cannot understand why older persons behave the way they do |
| I find it difficult to explain to older persons what is happening in situations which may upset them (e.g. showering, bathing or toileting) |
| **Factor 3 Balancing competing needs** |
| I have to balance the needs of the older person against the needs of his or her family |
| I have to balance the needs of the older person against the needs of other older persons |
| I have to prioritize on the basis of urgency rather than fairness or the needs of older persons |
| Older persons resist the care I want to provide |
| I have to balance the safety of older persons against their quality of life |
| **Factor 4 Balancing emotional involvement** |
| When an older person dies or has to move I feel as though I have lost a relative or close friend |
| I feel that older persons are highly dependent on me |
| I wish I knew more about older persons so that I could understand them better |
| I cannot stop thinking about older persons when I am away from work |
| **Factor 5 Lack of recognition** |
| I feel that my work is not valued by others |
| I want to do much more for older persons than my employers will allow |
| My employers do not appreciate the work I am doing |
| The families of older persons do not seem to understand how difficult it is to care for their relative |
