# Supplementary material for: Factors in the psychosocial work environment of staff are associated with satisfaction with care among older persons receiving home care services
Source: Health Soc Care Community. 2022 Sep 26;30(6):e6080–90. doi: 10.1111/hsc.14045 (PMC10087462; doi:10.1111/hsc.14045)
Supplement: Supplementary file 3 — Table S3 [file HSC-30-e6080-s001.docx]

Supplementary Table 3. Results from principal component analyses with varimax rotation on Questionnaire for Psychological and Social Factors at Work (QPS)

|  | Component | | | | | |
| --- | --- | --- | --- | --- | --- | --- |
|  | 1 | 2 | 3 | 4 | 5 | 6 |
| Item/question | Factor loadings | | | | | |
| **Support from manager** |  |  |  |  |  |  |
| Do your managers show interest in your health and wellbeing? | .616 |  |  |  |  |  |
| Are you encouraged to develop/improve? | .519 |  |  |  |  |  |
| Do you receive help and support from your manager, if you need it? | .734 |  |  |  |  |  |
| Does your manager show appreciation of your work? | .830 |  |  |  |  |  |
| Are you encouraged to participate in important decision making? | .813 |  |  |  |  |  |
| Do your managers help you to develop your skills? | .818 |  |  |  |  |  |
| **Work group climate** |  |  |  |  |  |  |
| Do you receive support from your colleagues? |  | .733 |  |  |  |  |
| Is the climate in the work group encouraging and supportive? |  | .616 |  |  |  |  |
| Is the climate in the work group relaxing and supportive? |  | .539 |  |  |  |  |
| Do you appreciate belonging to your work group? |  | .756 |  |  |  |  |
| Is your work group good at solving problems? |  | .815 |  |  |  |  |
| Is the communication within the department satisfactory? |  | .563 |  |  |  |  |
| **Sense of mastery** |  |  |  |  |  |  |
| Is your workload so unequally distributed that the work piles up? (reversed) |  |  | .643 |  |  |  |
| Is there too much to do? (reversed) |  |  | .733 |  |  |  |
| Are the work tasks too difficult? (reversed) |  |  | .762 |  |  |  |
| Are you assigned tasks that you need more training for? (reversed) |  |  | .565 |  |  |  |
| Are conflicting demands posed on you by two or more persons? (reversed) |  |  | .616 |  |  |  |
| Have you recently felt stressed? (reversed) |  |  | .649 |  |  |  |
| **Job control** |  |  |  |  |  |  |
| Can you influence the amount of workload you are given? |  |  |  | .589 |  |  |
| Can you set your own pace of work? |  |  |  | .757 |  |  |
| Can you decide when to take a break? |  |  |  | .786 |  |  |
| Can you influence decisions that are important for your work? |  |  |  | .580 |  |  |
| **Social environment at work** |  |  |  |  |  |  |
| Do rumours circulate about changes in the workplace? (reversed) |  |  |  |  | .482 |  |
| Is the climate in your work group rigid and rule-based? (reversed) |  |  |  |  | .536 |  |
| Have you noticed disturbing conflicts amongst colleagues? (reversed) |  |  |  |  | .713 |  |
| Have you noticed unequal treatment of women and men at your workplace? (reversed) |  |  |  |  | .706 |  |
| Have you noticed unequal treatment of colleagues of different ages? (reversed) |  |  |  |  | .620 |  |
| **Positive challenges** |  |  |  |  |  |  |
| Are your skills and knowledge useful in your work? |  |  |  |  |  | .796 |
| Does your job involve positive challenges? |  |  |  |  |  | .597 |
| Are there clearly defined goals for your work? |  |  |  |  |  | .453 |
| Do you know exactly what is expected of you at work? |  |  |  |  |  | .566 |
| Are you comfortable with your own ability to solve problems? |  |  |  |  |  | .540 |
