# Supplementary material for: Factors in the psychosocial work environment of staff are associated with satisfaction with care among older persons receiving home care services
Source: Health Soc Care Community. 2022 Sep 26;30(6):e6080–90. doi: 10.1111/hsc.14045 (PMC10087462; doi:10.1111/hsc.14045)
Supplement: Supplementary file 4 — Table S4. [file HSC-30-e6080-s003.docx]

Supplementary Table 4. Association between psychosocial work environment and work group related factors^1^ (n=16).

| **Psychosocial work environment variable** | **Explained variance** |
| --- | --- |
| Frustrated empathy | 0.713 |
| Difficulties understanding and interpreting | 0.490 |
| Balancing competing needs | 0.748 |
| Balancing emotional involvement | 0.753 |
| Lack of recognition | 0.877 |
| Total job strain | 0.741 |
| Support from manager | 0.743 |
| Work group climate | 0.806 |
| Sense of mastery | 0.805 |
| Job control | 0.815 |
| Social environment at work | 0.864 |
| Positive challenges | 0.373 |
| QPS total | 0.427 |

1) Linear regressions (n=16). Psychosocial work environment variables are dependent. Independent variables: average age, length of employment, proportion of women, Swedish as mother tongue, nursing assistant, permanent position, working full time, university education, education less than upper secondary, and care education.
